# A Modified Compound From Paeoniflorin, CP-25, Suppressed Immune Responses and Synovium Inflammation in Collagen-Induced Arthritis Mice

**DOI:** 10.3389/fphar.2018.00563

**Published:** 2018-06-07

**Authors:** Jingyu Chen, Ying Wang, Huaxun Wu, Shangxue Yan, Yan Chang, Wei Wei

**Affiliations:** Key Laboratory of Anti-inflammatory and Immune Medicine, Institute of Clinical Pharmacology, Anhui Medical University, Ministry of Education, Hefei, China

**Keywords:** paeoniflorin-6’-*O*-benzene sulfonate, collagen-induced arthritis, immune response, synovium inflammation, β2-adrenoceptor

## Abstract

Paeoniflorin-6’-*O*-benzene sulfonate (CP-25) is a modified paeoniflorin, which is the main bioactive component of total glucosides of peony. This study evaluated the anti-inflammatory and immunoregulatory effects of CP-25 in mice with collagen-induced arthritis (CIA) and the potential mechanisms underlying these effects. After the onset of CIA, mice were given CP-25 (17.5, 35, or 70 mg/kg) or methotrexate (MTX, 2.0 mg/kg). The arthritis index, swollen joint count, and joint and spleen histopathology were evaluated. T and B cell subsets were assayed using flow cytometry, while the proliferation of these cells and fibroblast-like synoviocytes (FLSs) were evaluated using the Cell Counting Kit-8. β2-adrenoceptor (β2-AR) expression was assayed using flow cytometry, immunohistochemistry, and western blotting. FLS migration and invasion were assayed using Transwells. CP-25 (35 or 70 mg/kg) attenuated the arthritis index and swollen joint count, alleviated joint and spleen histopathology, suppressed excessive T cell activation, and attenuated humoral immunity in CIA mice. CP-25 increased β2-AR expression on T cells, B cells, dendritic cells, and the synovium in CIA mice. CP-25 up-regulated the β2-AR agonist response and attenuated FLS activation; these effects may reflect CP-25-mediated reduction of β2-AR desensitization due to down-regulation of membrane G protein-coupled receptor kinase 2 expression. These results suggest that CP-25 suppressed immune responses and synovium inflammation in mice with CIA, effects that were associated with reduced β2-AR desensitization and the promotion of β2-AR signaling.

## Introduction

Rheumatoid arthritis (RA) is a chronic inflammatory autoimmune disease characterized by immoderate immune responses to self-antigens and synovium inflammation, which leads to the destruction of the joints (Pablos and Cañete, 2013; Rodeghero et al., 2013). Although the exact pathology is not clearly understood, RA is generally regarded to be a T cell-mediated autoimmune condition. In addition to its involvement in autoimmune diseases, the T cell-dependent immune response is essential for host defense against bacterial and viral infections (Boitard, 1992; Goodnow, 1997). During this response, activated immune cells interact each other, infiltrate in the synovium, and then result in chronic synovium inflammation and bone erosion in RA.

**FIGURE 1:**
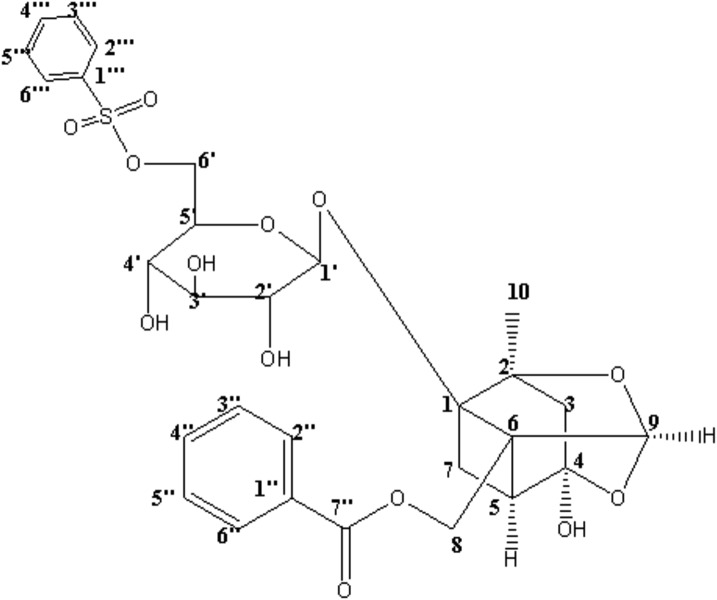
Structure of CP-25 (paeoniflorin-6’-*O*-benzene sulfonate).

CP-25 (paeoniflorin-6’-*O*-benzene sulfonate, Chinese patent number: ZL201210030616.4) is shown in **Figure 1**. This compound is derived from paeoniflorin (Pae), the main bioactive component of total glucosides of peony (TGP). As the first anti-inflammatory and immunoregulatory drug approved in China, TGP has been widely used for the treatment of RA. Compared with TGP, CP-25 has displayed improved absorption and distribution, slower clearance, a longer mean residence time, and moderate bioavailability in rats (Wang et al., 2016; Yang et al., 2016). Our previous studies showed that CP-25, as well as Pae and TGP, significantly inhibited the progression of experimental arthritis in rats by reducing inflammation, the immune response, and bone damage (Zheng and Wei, 2005; Zhu et al., 2005; Chang et al., 2016). Our previous studies also suggested that the anti-inflammatory and immunoregulatory activities of CP-25 may reflect its effects on G protein-coupled receptor (GPCR) signaling; this includes regulation of prostaglandin E2 receptors, including EP2 and EP4 (Xu et al., 2007; Chang et al., 2009, 2011b), G proteins (Jia et al., 2014), β-arrestin 2, and G protein-coupled receptor kinase 2 (GRK2) (Wang et al., 2011; Chen et al., 2012; Wu et al., 2012).

Recently, our data have shown that β2-adrenoceptor (β2-AR) signaling was weaker in rats with adjuvant-induced arthritis, as compared to control rats. Elevated GRK2 and decreased β2-AR expression were observed in the dendritic cells (DCs) of rats with adjuvant-induced arthritis, and suppression of β2-AR signaling may exacerbate arthritis-associated inflammation (Wu et al., 2016). Activation of β_2_-AR can produce an anti-inflammatory effect during RA; this is associated with an inhibition of the immune response and a suppression of synoviocyte proliferation (Wu et al., 2016). These findings indicate that attenuated β2-AR signaling may contribute to the development of RA, and targeting this receptor may therefore provide a potential strategy in the treatment of RA (Cobelens et al., 2002). The present study evaluated the effects of CP-25 on T cell-dependent immune responses and synovium inflammation in mice with collagen-induced arthritis (CIA), and investigated the potential mechanisms involved in the anti-inflammatory and immunoregulatory effects of this compound, with a focus on β2-AR signaling.

## Materials and Methods

### Animals and Cells

Sixty male DBA/1 mice (18 ± 2 g) (Shanghai SLAC Laboratory Animal Co., Ltd., Shanghai, China) were used in this study. All mice were maintained in the specific pathogen-free animal laboratory of Anhui Medical University (Hefei, China). The human RA fibroblast-like synoviocyte (FLS) cell line, MH7A, was purchased from the China Center for Type Culture Collection. All experiments were approved by the Ethics Review Committee for the Experimentation of the Institute of Clinical Pharmacology, Anhui Medical University.

### Reagents

Chick type II collagen and complete Freund’s adjuvant were purchased from Chondrex Inc. (Redmond, WA, United States); Gibco RPMI 1640 medium was from Life Technologies (Grand Island, NY, United States); and fluorescence-conjugated monoclonal antibodies to cluster of differentiation (CD) 3, CD4, CD62L, CD25, CD11c, CD19, CD138, interleukin (IL)-17, and interferon-γ (IFN-γ) were from BioLegend, Inc. (San Diego, CA, United States). Tumor necrosis factor-α (TNF-α) was purchased from PeproTech (Rocky Hill, NJ, United States). Fetal bovine serum (FBS) was purchased from Gibco (Carlsbad, CA, United States). The Cell Counting Kit-8 was from Dojindo Molecular Technologies, Inc. (Tokyo, Japan), and monoclonal antibodies raised against GRK2, β_2_-AR, the C-X-C chemokine receptor type 5, and β-actin were obtained from Abcam (Cambridge, United Kingdom). Transwell chambers (5.0 μm) were from Corning (Corning, NY, United States). CP-25 was supplied by the Chemistry Laboratory of Institute of Clinical Pharmacology of Anhui Medical University (Hefei, Anhui Province, China) and methotrexate was obtained from Shanghai Xinyi Pharmaceutical Co., Ltd. (Shanghai, China).

### CIA Induction and Treatment

Type II collagen was dissolved in 0.1 M acetic acid and emulsified with an equal volume of complete Freund’s adjuvant to produce a final concentration of 2 mg/ml before incubating overnight at 4°C. DBA/1 mice were injected twice intradermally with 0.1 ml of this emulsion (100 mg of type II collagen/mouse) at the back and the base of the tail. The day of the first immunization was defined as day 0, and the booster injection was administered into the back on day 21. Mice were divided into six groups (*n* = 10 per group): normal, CIA, CP-25 17.5 mg/kg, CP-25 35 mg/kg, CP-25 70 mg/kg, and MTX 2 mg/kg. After the onset of arthritis at day 28, mice were given CP-25 once daily by intragastric administration for 21 days (during the secondary arthritis phase). Methotrexate (2 mg/kg) was administered eight times (once every 3 days) intragastrically. Meanwhile, the normal and CIA animals were given an equal volume of vehicle.

### Evaluation of Arthritis

Arthritis global assessments and swollen joint counts were conducted every 3 days by two observers who were blinded to the treatment groups. The arthritis global assessment was based on observations of different parts of the mice: ear (0: no nodules or redness, 1: nodules and redness on one ear, 2: nodules and redness on both ears); nose (0: no connective tissue swelling or redness, 1: evident connective tissue swelling and redness); tail (0: no nodules or redness, 1: evident nodules and tail redness); and paw (0: no swelling or redness, 1: one front or hind paw with swelling and redness, 2: two paws with swelling and redness, 3: three paws with swelling and redness, 4: four paws with swelling and redness). The scores were tallied to form the arthritis global assessment, and the maximum value for each mouse was 8. The maximum swollen joint count for each mouse was 24 because each paw has five phalanx joints and one ankle or wrist joint (Chen et al., 2015).

### Histological Examination

The mice were anesthetized and killed at the end of the experimental period; the hind paws and spleen were fixed in 10% neutral-buffered formalin and then embedded in paraffin. The sections (5 μm) were stained with hematoxylin and eosin, and were examined microscopically. Joint and spleen histopathology was evaluated by two blinded observers. Four features were evaluated in each section of joint tissue: synovial proliferation, cellular infiltration, pannus formation, and cartilage erosion. The grading scheme consisted of ordinal categories ranging from 0 (no effect) to 3 (severe effect) (Chang et al., 2011a). The spleen was evaluated by examining the periarteriolar lymphoid sheaths, the lymphoid follicles, the marginal zone, the red pulp, and the total number of germinal centers in each section. The grading scheme consisted of ordinal categories ranging from 0 (no effect) to 4 (severe effect) (Germolec et al., 2004).

### Proliferation Assay of T Cells and B Cells

A single-cell suspension was prepared from the thymus and spleen by mechanical dissociation of the tissue through nylon mesh. These cells were suspended in RPMI-1640 medium at a concentration of 1 × 1010 cell/l. Thymocytes (100 μl) and 100 μl Con A (final concentration of 5 mg/l) or splenocytes (100 μl) and 100 μl LPS (final concentration of 4 mg/l) were added into 96-well flat-bottomed culture plates. The cultures were incubated at 37°C in 5% CO_2_ for 48 h. At the end of the incubation period, Cell Counting Kit reagent (10 μl) was added to each well, and cells were incubated at 37°C for an additional 2 h. The absorbance was measured at a wavelength of 450 nm on microplate reader.

### Preparation of Mononuclear Cells

A single-cell suspension was prepared from the spleen by mechanical dissociation of the tissue through nylon mesh. Mononuclear cells were purified from the gradient interphase. The cells were then washed with phosphate-buffered saline three times and suspended in RPMI 1640 medium at a concentration of 1 × 10^7^ cells/ml for T-cell and B-cell subset assays.

### Flow Cytometry Analyses of T Cells, B Cells, and DCs

To assay the T cell and B cell subsets, and the expression of β2-AR on T cells, B cells, and DCs, fluorescence-conjugated antibodies were added to the single-cell spleen suspension (100 μl). After gentle mixing, the samples were incubated for 20 min at 4°C prior to analysis using flow cytometry.

### Immunohistochemical Analysis of β2-AR Expression in the Synovium

The avidin-biotin method was applied to paraffin-embedded blocks of formalin-fixed joint tissue, which were cut into 4-μm sections, deparaffinized with xylene, and blocked with hydrogen peroxide. Sections were incubated with an antibody raised against the β2-AR (1:50 dilution) in a humid chamber at 4°C overnight. After washing with phosphate-buffered saline, the sections were incubated an avidin-coupled secondary antibody, followed by a substrate an avidin-coupled secondary antibody, followed by a substrate for 30 min to visualize the β2-AR distribution. Five microscopic fields were inspected in each section. The average optical density of the staining was analyzed using the JEDA 801D Morphology Image Analysis System.

### The RA-Derived Fibroblast-Like Synoviocyte Cell Line Treatment

The RA-derived FLS cell line, MH7A, was incubated in Dulbecco’s modified Eagle’s medium containing 10% FBS at 37°C in an incubator with 5% CO_2_, and pretreated with TNF-α (2 ng/ml) or TNF-α (2 ng/ml) plus CP-25 (0.1, 1, or 10 μM) for 24 h. Then isoproterenol (ISO) was added at a concentration of 1 μM and the cells were incubated for 6 h prior to examining their proliferation, migration, and invasion.

### Analysis of Cell Proliferation

Cell viability was assayed using the Cell Counting Kit-8. At the end of the incubation period, Cell Counting Kit reagent (10 μl) was added to each well, and cells were incubated at 37°C for an additional 2 h. The absorbance was measured at a wavelength of 450 nm on microplate reader.

### Analysis of MH7A Cell Migration

MH7A cells were treated as described before trypsinization and suspension in serum-free Dulbecco’s modified Eagle’s medium at a density of 6 × 10^4^ cells/ml. One-hundred microliters of the MH7A suspension was added to an upper Transwell chamber, and 600 μl Dulbecco’s modified Eagle’s medium supplemented with 20% FBS was added to the lower chamber. After incubation for 24 h at 37°C, non-migrating MH7A cells (on the upper face of the filter) were removed using cotton swabs. MH7A cells that had migrated to below the filter were stained with 0.1% crystal violet for 10 min, and washed twice with phosphate-buffered saline. The stained MH7A cells were then observed using an optical microscope (magnification ×100) and counted; the mean number of cells in five randomly chosen fields was then calculated.

### Analysis of GRK2 and β2-AR Expression by Western Blotting

MH7A cells were treated as indicated before lysis in cell lysis buffer and protein preparation; denatured protein was separated by 10% sodium dodecyl sulfate-polyacrylamide gel electrophoresis and transferred electrophoretically to a polyvinylidene fluoride membrane. After incubating with blocking buffer (0.05% Tween 20/phosphate-buffered saline with 5% non-fat milk) at 37°C for 2 h, the membrane was incubated overnight at 4°C with a primary antibody to either GRK2 or β2-AR at a final dilution of 1:1000. After incubating with the appropriate goat anti-rabbit antibody at 37°C for 2 h, immunodetection was carried out using enhanced chemiluminescence, according to the manufacturer’s instructions. Equivalent protein loading and transfer efficiency were verified by staining for β-actin.

### Statistical Analysis

Data were expressed as the mean ± the standard deviation (SD). Comparisons between multiple groups were made using analysis of variance, and comparisons between two groups were performed using Student’s *t*-test; *P*-values < 0.05 were considered to be significant.

## Results

### CP-25 Attenuated Arthritis Signs in CIA Mice

**FIGURE 2:**
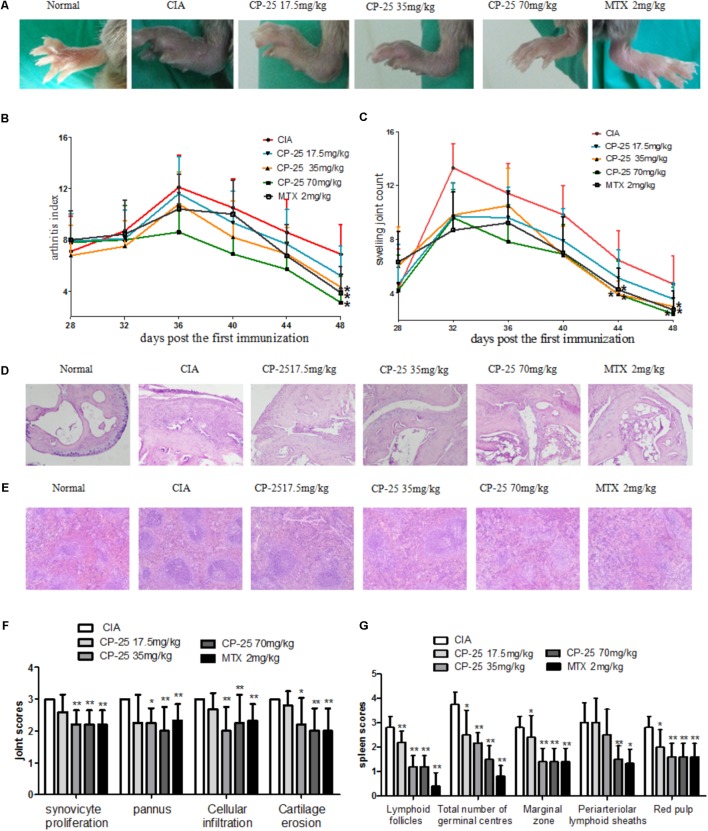
CP-25 alleviated the severity of arthritis and joint and spleen histopathology in CIA mice. **(A)** Joint swelling (d44, 35 mg/kg: *t* = 2.457, *P* < 0.05; 70 mg/kg: *t* = 3.306, *P* < 0.05) (d48, 35 mg/kg: *t* = 2.561, *P* < 0.05; 70 mg/kg: *t* = 2.005, *P* < 0.05); **(B)** arthritis index (d48, 35 mg/kg: *t* = 2.161, *P* < 0.05; 70 mg/kg: *t* = 2.788, *P* < 0.05); **(C)** joint swelling counts (35 mg/kg: *t* = 2.294, *P* < 0.05; 70 mg/kg: *t* = 3.011, *P* < 0.05). Data are expressed as the mean ± SD, with 10 animals in each group. Photomicrographs of **(D)** the mouse joint and **(E)** the mouse spleen (original magnification ×100, hematoxylin and eosin stain). **(F)** Evaluation of joint inflammation scores. Synoviocyte proliferation (35 mg/kg: *t* = 4.000, *P* < 0.01; 70 mg/kg: *t* = 4.000, *P* < 0.01), cellular infiltration (35 mg/kg: *t* = 3.163, *P* < 0.01; 70 mg/kg: *t* = 4.000, *P* < 0.01), pannus formation (35 mg/kg: *t* = 4.000, *P* < 0.05; 70 mg/kg: *t* = 6.000, *P* < 0.01), and cartilage erosion (35 mg/kg: *t* = 3.204, *P* < 0.05; 70 mg/kg: *t* = 3.295, *P* < 0.01). **(G)** Evaluation of spleen inflammation scores. Cellularity of the periarteriolar lymphoid sheaths (70 mg/kg: *t* = 6.608, *P* < 0.01), lymphoid follicles (35 mg/kg: *t* = 5.657, *P* < 0.01; 70 mg/kg: *t* = 5.657, *P* < 0.01), marginal zone (35 mg/kg: *t* = 4.427, *P* < 0.01; 70 mg/kg: *t* = 4.427, *P* < 0.01), the total number of germinal centers (35 mg/kg: *t* = 2.936, *P* < 0.01; 70 mg/kg: *t* = 4.782, *P* < 0.01), and red pulp (35 mg/kg: *t* = 3.795, *P* < 0.01; 70 mg/kg: *t* = 3.795, *P* < 0.01). Data are expressed as the mean ± SD, with six animals in each group. ^∗^*P* < 0.05, ^∗∗^*P* < 0.01, vs. CIA mice.

The onset of the arthritis of DBA/1 mice appeared on day 28 after the first immunization (at day 0). To evaluate the therapeutic effect of CP-25 on CIA mice, CP-25 was administered to CIA mice from day 28 to day 48, and the arthritis index and swollen joint count were assessed. The results revealed significantly swollen joint counts in CIA mice, as compared with normal mice (**Figure 2A**). Administration of CP-25 (35 or 70 mg/kg) significantly decreased the arthritis index (**Figure 2B**) and swollen joint count (**Figure 2C**).

### CP-25 Alleviated Joint and Spleen Histopathology in CIA Mice

The main joint histopathology in CIA mice was characterized by the proliferation of synoviocytes, the appearance of pannus, and bone erosion. CP-25 (35 or 70 mg/kg) significantly attenuated synoviocyte proliferation, cellular infiltration, pannus formation, and cartilage erosion in CIA mice (**Figures 2D,F**). The white pulp of the spleen is filled with lymphocytes and is the site where T cells and B cells undergo activation, proliferation, and differentiation; lymphoid white pulp hyperplasia was the main damage observed in the spleen of CIA mice. The histopathology results showed that administration of CP-25 (35 or 70 mg/kg) alleviated cellularity of the periarteriolar lymphoid sheaths, lymphoid follicles, marginal zone, the total number of germinal centers, and red pulp (**Figures 2E,G**).

### CP-25 Suppressed the T Cell Immune Response in CIA Mice

**FIGURE 3:**
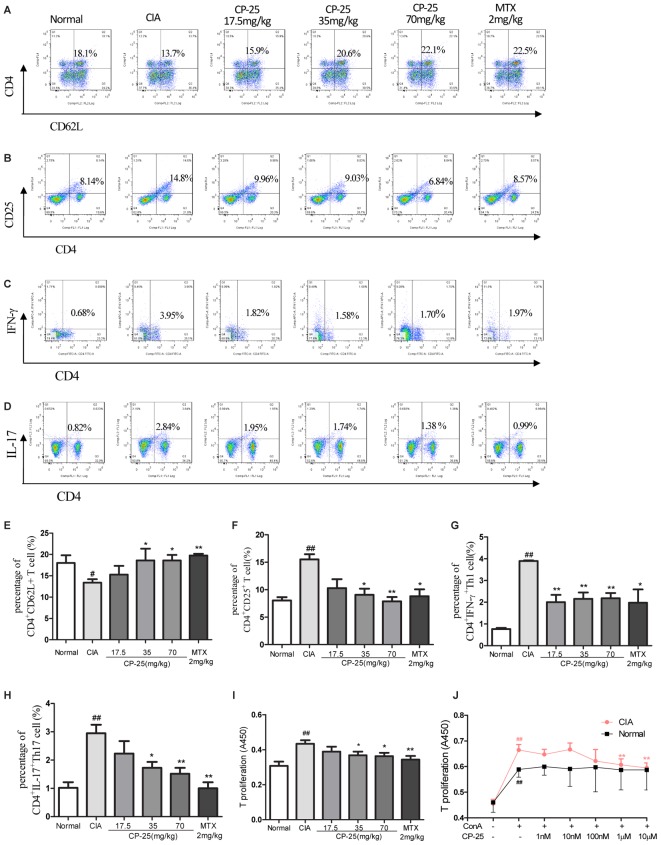
CP-25 suppressed excessive T cell activation and the generation of effector T cells in CIA mice. **(A,E)** Naïve T cell (35 mg/kg: *t* = –1.820, *P* < 0.05; 70 mg/kg: *t* = –3.384, *P* < 0.05); **(B,F)** activated T cell (35 mg/kg: *t* = 4.361, *P* < 0.05; 70 mg/kg: *t* = 6.250, *P* < 0.01); **(C,G)** Th1 (35 mg/kg: *t* = 5.920, *P* < 0.01; 70 mg/kg: *t* = 7.004, *P* < 0.01); **(D,H)** Th17 (35 mg/kg: *t* = 5.920, *P* < 0.01; 70 mg/kg: *t* = 7.004, *P* < 0.01). Data are expressed as the mean ± SD, with six animals in each group. ^#^*P* < 0.05, ^##^*P* < 0.01 vs. Normal; ^∗^*P* < 0.05, ^∗∗^*P* < 0.01 vs. CIA. **(I)** T cell proliferation (35 mg/kg: *t* = 2.282, *P* < 0.05; 70 mg/kg: *t* = 2.563, *P* < 0.05). Data are expressed as the mean ± SD, with six animals in each group. ^##^*P* < 0.01 vs. Normal; ^∗^*P* < 0.05, ^∗∗^*P* < 0.01 vs. CIA. **(J)** T cell proliferation *in vitro* (1 μM: *t* = 3.649, *P* < 0.01; 10 μM: *t* = 4.757, *P* < 0.01). Data are expressed as the mean ± SD. ^##^*P* < 0.01 vs. control; ^∗∗^*P* < 0.01 vs. LPS. The results are representative of at least three independent experiments.

To evaluate the activation of T cells, the subsets of naïve T cells (CD4^+^CD62L^+^) and activated T cells (CD4^+^CD25^+^) were assayed in the mouse spleen. In CIA mice, a lower percentage of naïve T cells (**Figures 3A,E**) and an increased percentage of activated T cells (**Figures 3B,F**) was observed, as compared with the normal mice. Administration of CP-25 (35 or 70 mg/kg) resulted in a decreased number of activated T cells (**Figures 3B,F**) and an increased level of naïve T cells (**Figures 3A,E**) in the spleen of CIA mice. Th1 (CD4^+^IFN-γ^+^) and Th17 (CD4^+^IL-17^+^) are the main T cells involved in the pathological changes observed in CIA mice, including synovium inflammation. In the current study, we analyzed the percentages of Th1 and Th17 cells. The results revealed that CP-25 (35 or 70 mg/kg) down-regulated the percentages of Th1 (**Figures 3C,G**) and Th17 (**Figures 3D,H**) in the spleens of CIA mice. The effect of CP-25 on T cell proliferation was evaluated both *in vivo* and *in vitro. In vivo*, CP-25 (35 or 70 mg/kg) significantly suppressed T cell proliferation (**Figure 3I**). Consistent with this result, CP-25 (1 μM or 10 μM) significantly suppressed the *in vitro* proliferation of T cells from CIA mice (*P* < 0.01), while not affecting the proliferation of normal T cells (**Figure 3J**).

### CP-25 Attenuated Humoral Immunity in CIA Mice

**FIGURE 4:**
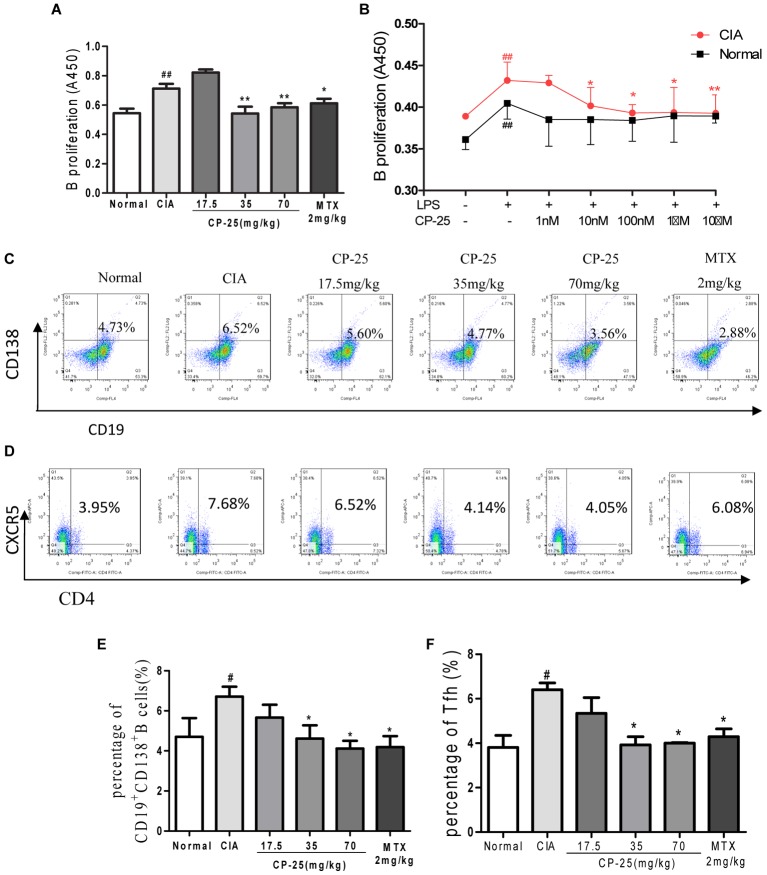
CP-25 attenuated B cell activation in CIA mice. **(A)** B cell proliferation (35 mg/kg: *t* = 2.961, *P* < 0.01; 70 mg/kg: *t* = 2.973, *P* < 0.01). Data are expressed as the mean ± SD, with six animals in each group. ^##^*P* < 0.01 vs. Normal; ^∗^*P* < 0.05, ^∗∗^*P* < 0.01 vs. CIA. **(B)** B cell proliferation *in vitro* (10 nM: *t* = 2.130, *P* < 0.05; 100 nM: *t* = 3.020, *P* < 0.05; 1 μM: *t* = 2.250, *P* < 0.05; 10 μM: *t* = 2.735, *P* < 0.05). Data are expressed as the mean ± SD. ^##^*P* < 0.01 vs. control; ^∗^*P* < 0.05, ^∗∗^*P* < 0.01 vs. LPS. The results are representative of at least three independent experiments. **(C,E)** CD19^+^CD138^+^ B cells (35 mg/kg: *t* = 2.538, *P* < 0.05; 70 mg/kg: *t* = 4.157, *P* < 0.05); **(D,F)** T follicular helper cells (35 mg/kg: *t* = 4.872, *P* < 0.05; 70 mg/kg: *t* = 7.790, *P* < 0.05). Data are expressed as the mean ± SD, with six animals in each group. ^#^*P* < 0.05 vs. Normal; ^∗^*P* < 0.05 vs. CIA.

B cell proliferation, the plasma B cell subset, were assayed to investigate the effects of CP-25 on humoral immunity. These analyses showed that B cell proliferation was suppressed by 35 or 70 mg/kg CP-25 *in vivo* (**Figure 4A**) and by 10 nM, 100 nM, or 1 μM CP-25 *in vitro*; however, CP-25 had no effect on the proliferation of normal B cells *in vitro* (**Figure 4B**). Plasma B cells are the major source of antibody production, and their secretion of pathogenic autoantibodies can maintain a chronically autoreactive environment. The percentage of CD19^+^CD138^+^ plasma B cells was down-regulated in mice treated with 35 or 70 mg/kg CP-25 (**Figures 4C,E**). T follicular helper cells are responsible for B cell activation, proliferation, and differentiation, and for the development of plasma B cells. The present study revealed that CP-25 (35 or 70 mg/kg) down-regulated the percentage of T follicular helper cells (**Figures 4D,F**) in the spleens of CIA mice.

### CP-25 Increased Immune Cells and Synovium β2-AR Expression in CIA Mice

**FIGURE 5:**
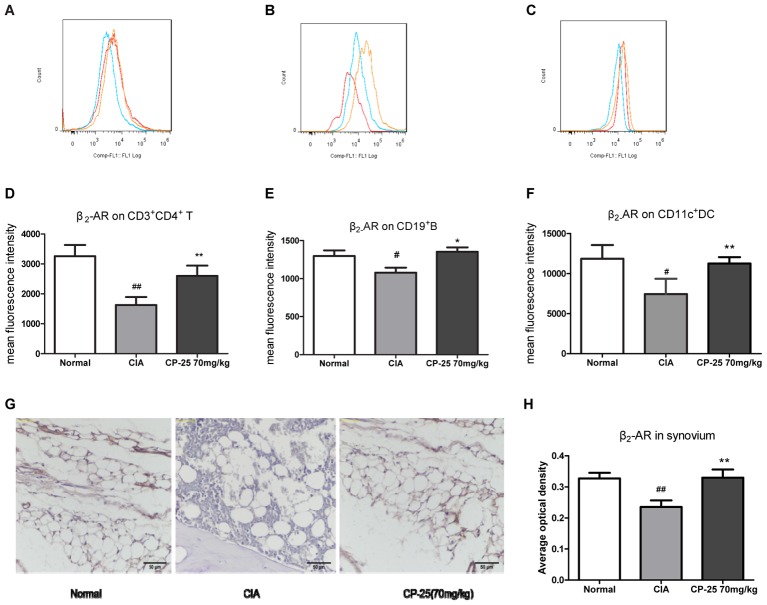
CP-25 increased β2-AR expression by immune cells and synovium in CIA mice. β2-AR expression on **(A,D)** CD3^+^CD4^+^ T cells (70 mg/kg: *t* = –6.691, *P* < 0.01); **(B,E)** CD19^+^ B cells (70 mg/kg: *t* = –5.349, *P* < 0.05); **(C,F)** CD11c^+^ DCs (70 mg/kg: *t* = –4.130, *P* < 0.01). **(G)** Immunohistochemical analysis of the expression of β2-AR in the synovium; original magnification ×200. **(H)** Average optical density analysis of β2-AR expression (70 mg/kg: *t* = –4.833, *P* < 0.01). Data are expressed as the mean ± SD, with six animals in each group. ^#^*P* < 0.05, ^##^*P* < 0.01 vs. Normal; ^∗^*P* < 0.05, ^∗∗^*P* < 0.01 vs. CIA.

Collagen-induced arthritis mice showed reduced β2-AR expression on the membranes of CD3^+^CD4^+^ T cells (**Figures 5A,D**), of CD19^+^ B cells (**Figures 5B,E**), on CD11c^+^ DCs (**Figures 5C,F**), and in the synovium (**Figures 5G,H**). Administration of CP-25 attenuated this effect on the membrane expression of β2-AR.

### CP-25 Up-Regulated β2-AR Response to Agonist by Decreasing β2-AR Desensitization

**FIGURE 6:**
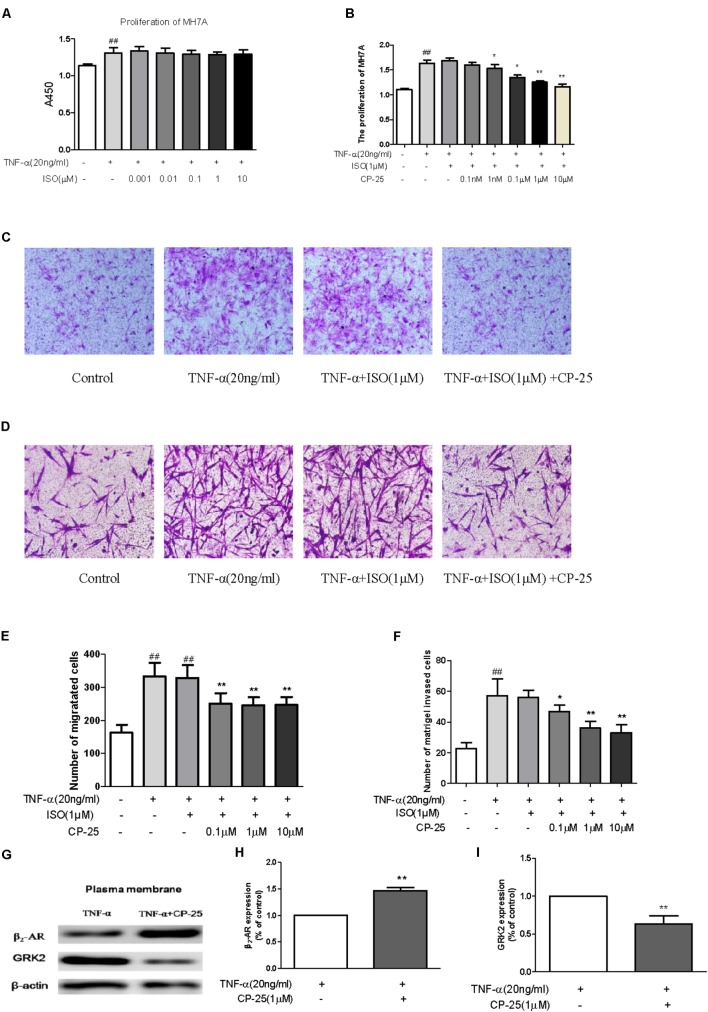
CP-25 down-regulated excessive synoviocyte activation by decreasing GRK2-mediated β2-AR desensitization. MH7A **(A,B)** proliferation (1 nM: *t* = 1.067, *P* < 0.05; 0.1 μM: *t* = 3.584, *P* < 0.01; 1 μM: *t* = 5.768, *P* < 0.01; 10 μM: *t* = 5.685, *P* < 0.01), **(C,E)** migration (0.1 μM: *t* = 5.268, *P* < 0.01; 1 μM: *t* = 6.141, *P* < 0.01; 10 μM: *t* = 6.070, *P* < 0.01), and **(D,F)** invasion (0.1 μM: *t* = 2.514, *P* < 0.05; 1 μM: *t* = 5.477, *P* < 0.01; 10 μM: *t* = 5.691, *P* < 0.01). Data are expressed as the mean ± SD. ^##^*P* < 0.01 vs. control; ^∗^*P* < 0.05, ^∗∗^*P* < 0.01 vs. TNF + ISO. The results are representative of at least three independent experiments. **(G–I)** Expression of β2-AR (*t* = –12.745, *P* < 0.01) and GRK2 (*t* = 5.986, *P* < 0.01). The results are representative of at least three independent experiments and data are expressed as the mean ± SD. ^∗∗^*P* < 0.01 vs. TNF.

We next explored whether this up-regulation of β2-AR affected cellular responses to a β2-AR agonist, and whether this was responsible for the anti-inflammatory and immunoregulatory effects of CP-25 in CIA mice. The effects of ISO and/or CP-25 on the proliferation, migration, and invasion of TNF-α-induced MH7A were investigated; this represented an *in vitro* model of RA-associated inflammation. ISO had no effect on MH7A proliferation, migration, or invasion; however, exposure to both CP-25 and ISO suppressed the proliferation (**Figures 6A,B**), migration (**Figures 6C,E**), and invasion of MH7A cells (**Figures 6D,F**). These results suggested that CP-25 up-regulated β2-AR response to ISO in MH7A. Receptor desensitization is involved in the regulation of β2-AR membrane expression. GRK2 is the main kinase subtype responsible for β2-AR desensitization, which regulates the ligand response of this receptor. To investigate the mechanism underlying the decrease in membrane β2-AR expression by FLS, both GRK2 and β2-AR membrane expression were assayed. The results showed that CP-25 up-regulated membrane expression of β2-AR and down-regulated membrane expression of GRK2 on synoviocytes (**Figures 6G–I**).

## Discussion

We previously reported that CP-25 exerted anti-inflammatory and anti-osteoclastic effects in rats with adjuvant-induced arthritis (Chang et al., 2016). In the present study, the effects of CP-25 on the development of arthritis, as well as on the T cell-mediated immune response and synovium inflammation, were investigated in CIA mice. Activated T cells play a pivotal role in RA-associated inflammation by producing inflammatory cytokines, which induce immune responses and synovium inflammation. The inhibition of T cell activation has been identified as a potential clinical treatment for RA (Dulic et al., 2017). The present study found that *in vivo* administration of CP-25 to CIA mice up-regulated naïve T cells and down-regulated activated T cells, while T cell proliferation was suppressed both *in vivo* and *in vitro*. From these data, we infer that the anti-inflammatory effects of CP-25 may be related to the down-regulation of excessive T cell activation. It was interesting that CP-25 had a significant impact on the proliferation of T cells and B cells from CIA mice, but had no effect on the *in vitro* proliferation of normal T cells and B cells. This suggested that CP-25 may only regulate this excessive immune response, without influencing the normal immune response.

Subsets of T cells, including Th1 and Th17, infiltrate the synovium and secrete effector cytokines such as IFN-γ, IL-17, and IL-23; this results in chronic synovium inflammation, synoviocyte hyperplasia, and bone destruction in joints. Th17 (CD4^+^IL-17^+^) is the main Th cell subtype involved in the pathological changes observed in CIA mice, which include synovium inflammation and joint damage. Th17 cells and the characteristic cytokine, IL-17, are more highly expressed at sites of inflammation and in the peripheral circulation of patients with RA than in healthy controls. IL-17A induced synoviocytes to produce proinflammatory cytokines (such as IL-1β, TNF, and IL-6) and chemokines (such as IL-8, and CC chemokine ligands 2 and 20), which promoted the migration of neutrophils, monocytes, Th17 cells, and DCs into the synovium. Co-culture of Th17 cells with synoviocytes from early RA patients promoted the release of proinflammatory cytokines and matrix metalloproteases. In addition, IL-17 and IL-23 promoted the expression of receptor activator of NFκ-B and receptor activator of nuclear NFκ-B ligand, thereby inducing osteoclastogenesis (Zhu and Qian, 2012). Our previous study showed that CP-25 down-regulated the production of IL-17 and of the Th17-associated transcription factor, RAR-related orphan receptor gamma (Chang et al., 2016). Consist with these data, the present study showed that CP-25 down-regulated the percentage of Th17 cells in the spleen of CIA mice. These results suggest that the anti-inflammatory and immunoregulatory effects of CP-25 on CIA mice may be due to the down-regulation of Th17.

The T cell-dependent antibody response is indispensable for host protection against viral and bacterial infections, as well as driving the development of autoimmune diseases such as RA. During this process, Th cells and B cells interact and secrete cytokines and antibodies, respectively, which result in immune protection or tissue damage. T follicular helper cells are the key Th subtype that activates B cells, inducing plasma differentiation and antibody production. In the current study, CP-25 down-regulated B cell proliferation, CD19^+^CD138^+^ plasma B cells, and the CD4^+^C-X-C chemokine receptor type 5^+^ T follicular helper subset. The crosstalk between T cells and B cells is definitely involved in auto-antibody production in various models of RA. The spleen white pulp is filled with lymphocytes and is the site where T cells and B cells undergo activation, proliferation, and differentiation. T follicular helper cells are crucial for the production of auto-antibodies in the germinal center of the white pulp. Consistent with the above findings, the histopathology results showed that CP-25 alleviated white pulp hyperplasia, reduced the scores relating to the lymph node, germinal center, and marginal zone, and down-regulated germinal center scores in the spleen. In the pathogenesis of autoimmune diseases, immune cells are abnormally activated. Our previous study found that CP-25 inhibited activated human B cells by regulating B-cell activating factor and TNF-α signaling. Compared with the effects of rituximab and etanercept, CP-25 showed modest activity, suggesting that CP-25 may provide a promising approach to the soft regulation of inflammation (Zhang et al., 2017).

Our previous studies indicated that Pae suppressed fibroblast synoviocyte proliferation and the production of pro-inflammation cytokines via the regulation of EP4 signaling (Xu et al., 2007; Chang et al., 2009, 2011b). *In vitro*, CP-25 regulated DC maturation via the prostaglandin E2 signaling pathway (Li et al., 2015). These data suggested that the anti-inflammatory and immunoregulatory effects of CP-25 may be due to the regulation of GPCR signaling. In the current study, we found that CP-25 up-regulated the membrane expression of β2-AR on immune cells and synoviocytes from CIA mice *in vivo*. The results also revealed that although ISO had no effects on MH7A proliferation, migration, or invasion, these activities were suppressed by ISO in MH7A cells pretreated with CP-25; this suggested that CP-25 up-regulated the response of RA synoviocytes to ISO.

The β2-AR GPCR is widely expressed in a range of cell types, including immune cells. A number of studies have demonstrated the complex and time-dependent immunomodulatory effects of this receptor. *In vivo*, the administration of a β2-AR agonist to rats with adjuvant-induced arthritis prior to or at the time of adjuvant challenge (Coderre et al., 1990) resulted in disease exacerbation, while β2-AR agonist treatment at or after disease onset reduced disease severity (Malfait et al., 1999; Lubahn et al., 2004). β2-AR activation produces an anti-inflammatory effect in RA, including an inhibition of the immune response and a suppression of synoviocyte proliferation (Wu et al., 2016). Both *in vivo* and *in vitro*, β2-AR agonists significantly inhibit immune responses by down-regulating the activities of T cells and B cells (Kohm and Sanders, 2001). Activation of the β2-AR suppresses IL-2 production and inhibits T cell proliferation, which is required for clonal expansion. In synoviocytes, noradrenaline inhibits the secretion of proinflammatory cytokines such as IL-8 and TNF-α (Miller et al., 2002; Riepl et al., 2010). Attenuated β2-AR signaling contribute to the development of RA, and targeting β_2_-AR may be a potential strategy in the treatment of RA. The density and affinity of β2-ARs on peripheral blood mononuclear cell and synovial fluid lymphocytes were decreased in RA, and these changes showed a negative correlation with disease severity (Baerwald et al., 1997; Wahle et al., 2001). This decline in β2-AR expression attenuated the inhibitory effects of catecholamines on lymphocyte proliferation (Baerwald et al., 1999). These results indicated that the anti-inflammatory and immunoregulatory effects of CP-25 may be due to β2-AR activation.

Desensitization, as an important character of GPCR, is involved in the regulation of β_2_-AR membrane expression and then resulted in regulating the signaling of GPCR. GRK2 is involved in β2-AR desensitization and thus regulates the ligand response of this receptor. The present study found that CP-25 down-regulated the membrane expression of GRK2 and up-regulated that of the β2-AR in synoviocytes. These results suggest that a reduction of GRK2-mediated β2-AR desensitization may be responsible for the increased expression of β2-AR on the synoviocyte membrane, which may increase their response to ISO.

## Conclusion

CP-25 significantly attenuated the histopathological changes observed in the joints and spleens of CIA mice. CP-25 suppressed the proliferation of T and B cells, up-regulated the percentage of naïve T cells, and down-regulated the percentage of activated T cells, Th1, Th17, T follicular helper, and plasma B cells in CIA mice. *In vivo*, CP-25 up-regulated β2-AR expression by T cells, B cells, DCs, and synovium in CIA mice. *In vitro*, a β2-AR agonist (ISO) had no effect on RA synoviocyte proliferation, migration, or invasion, while treatment with both CP-25 and ISO suppressed these activities. CP-25 up-regulated synoviocyte membrane expression of the β2-AR, and down-regulated that of GRK2. These results suggest that CP-25 suppressed immune responses and synovium inflammation in CIA mice by reducing β2-AR desensitization and thus promoting β2-AR signaling.

## Author Contributions

WW and JC participated in the research design and contributed to the writing of the manuscript. JC and YW conducted the experiments and performed the data analysis. HW, SY, and YC contributed new reagents or analytical tools.

## Conflict of Interest Statement

The authors declare that the research was conducted in the absence of any commercial or financial relationships that could be construed as a potential conflict of interest.
